# *In-vivo* shift of the microbiota in oral biofilm in response to frequent sucrose consumption

**DOI:** 10.1038/s41598-018-32544-6

**Published:** 2018-09-21

**Authors:** Annette Carola Anderson, Michael Rothballer, Markus Jörg Altenburger, Johan Peter Woelber, Lamprini Karygianni, Ilias Lagkouvardos, Elmar Hellwig, Ali Al-Ahmad

**Affiliations:** 1 Department of Operative Dentistry and Periodontology, Faculty of Medicine, Albert-Ludwigs- University, Freiburg, Germany; 2 Institute of Network Biology, Helmholtz Zentrum München, German Research Center for Environmental Health, Neuherberg, Germany; 3 Clinic of Preventive Dentistry, Periodontology and Cariology, Center of Dental medicine, University of Zürich, Zürich, Switzerland; 4 ZIEL - Institute for Food and Health, Core Facility Microbiome/NGS, Technical University of Munich, Freising, Germany

## Abstract

Caries is associated with shifts of microbiota in dental biofilms and primarily driven by frequent sucrose consumption. Data on environmentally induced *in vivo* microbiota shifts are scarce therefore we investigated the influence of frequent sucrose consumption on the oral biofilm. Splint systems containing enamel slabs were worn for 3 × 7 days with 7-day intervals to obtain oral biofilm samples. After a three-month dietary change of sucking 10 g of sucrose per day in addition to the regular diet, biofilm was obtained again at the end of the second phase. The microbiota was analysed using Illumina MiSeq amplicon sequencing (v1-v2 region). In addition, roughness of the enamel surface was measured with laser scanning microscopy. The sucrose phase resulted in significant differences in beta-diversity and significantly decreased species richness. It was marked by a significant increase in abundance of streptococci, specifically *Streptococcus gordonii*, *Streptococcus parasanguinis* and *Streptococcus sanguinis*. Enamel surface roughness began to increase, reflecting initial impairment of dental enamel surface. The results showed that frequent sucrose consumption provoked compositional changes in the microbiota, leading to an increase of non-mutans streptococci, hence supporting the extended ecological plaque hypothesis and emphasizing the synergy of multiple bacterial species in the development of caries.

## Introduction

Caries, or tooth decay, presents a major health concern worldwide, affecting 2.4 billion people, resulting in high treatment costs^1^. The development of caries involves acids as bacterial fermentation products of the oral biofilm, and results in demineralisation of dental enamel and dentin^2,3^. According to the extended ecological plaque hypothesis, changes in the local environment, e.g. frequent carbohydrate availability, assumedly favor certain representatives of the oral biofilm and thus lead to a distinct shift in the microbiota composition towards a higher proportion of acidogenic and acid-tolerant species^4–6^.

Even though this hypothesis is widely accepted, the detailed microbial etiology remains a main focus of caries research and has not yet been fully clarified. This is also due to the fact that the oral cavity is a habitat for a resident microbiome which is known to vary substantially between individuals and therefore might also include individual low-abundant species. The latter can cause disease, when, due to favorable environmental conditions, they are given the chance to proliferate and outcompete other members of the oral biofilm^4^.

Formerly, *Streptococcus mutans* and a few other species had been considered the main cariogenic pathogens. However, during the last decades studies of the caries-associated microbiota have revealed a more complex composition of cariogenic plaque^2,7–10^. It has also been shown that dental plaque of healthy subjects can contain high proportions of *S. mutans* while at the same time caries can occur in the absence of *S. mutans*^6,11^. Studies confirmed that cariogenic plaque most often contains elevated levels of *S. mutans*, *Streptococcus sobrinus* and different *Lactobacillus* species^6^. Nevertheless, various other bacterial species have been shown to be associated with different stages of caries, e.g. non-mutans streptococci, such as *S. salivarius*, *S. parasanguinis* and also *Actinomyces* spp. have been linked to the initiation of caries^7,12,13^, whereas *Veillonella*, *Propionibacterium*, *Lactobacillus*, *Bifidobacterium*, *Atopobium* species have been associated with its progression^7^.

Analyzing the microbiota in oral health and disease has been performed with culture methods as well as molecular techniques, e.g. sequencing of 16S rRNA gene clone libraries^12,14–16^ and recently with high-throughput sequencing, particularly pyrosequencing and Illumina sequencing^17–21^. These approaches seem especially useful considering that an estimated 50% of the oral taxa are considered as yet uncultivated^3^.

Among the dietary factors, frequent consumption of fermentable carbohydrates has been shown to have a strong influence on the ecology of the oral biofilm and the development of caries^2,22^. Abundant evidence has demonstrated that frequent intake of sugars, especially sucrose, lowers the pH and disequilibrates the demineralization and remineralization cycles, resulting in net demineralization of dental enamel^2,23^. To date, numerous studies have used questionnaires tracking the diet of studied subjects, or artificial biofilm model systems to analyse the influence of sucrose and other carbohydrates on plaque bacteria^24–29^. However, these studies cannot fully mirror or reproduce the actual situation in the oral cavity. In particular, model systems that use artificial growth media will always lose a certain proportion of the original taxonomic diversity, even with complex growth media, due to the fastidious growth conditions of certain oral taxa. The majority of studies deal with severe caries, e.g. severe early childhood caries or progressed stages of carious lesions^8,10,30–32^, yet only few studies examine the early stages and the onset of caries^7,12,33^, or extend knowledge beyond children and deciduous teeth towards adults with permanent teeth.

To our knowledge, reports on the changes of the dental plaque microbiota under defined dietary conditions in an *in-vivo* situation are not available. Therefore we used an *in-situ* splint system to sample the dental plaque of eleven adults before and at the end of a 3-month long dietary change involving frequent sucrose consumption by sucking small pieces of 2 g rock candy five times daily. The dental biofilm was allowed to develop on enamel slabs over the course of seven days. Even though most people perform at least an average oral hygiene twice a day, studies have shown that plaque removal by tooth brushing and flossing is usually incomplete and certain surfaces tend to be neglected^34^. Therefore, dental plaque can remain for extended periods of time and transform into cariogenic plaque.

This sampling approach was combined with a high-throughput sequencing technique for the analysis of the plaque microbiota, and measurements of the surface roughness of the dental enamel after the biofilm removal using a Keyence 3D Laserscanning Microscope VK-X210. The questions we aimed to answer were the following: How does the plaque microbiota composition change in an *in-vivo* situation during frequent carbohydrate exposure over an extended time period? What kind of impact did the changed oral biofilm have on the underlying enamel surface?

## Results

### Dietary assessment of the study participants

**Figure 1:**
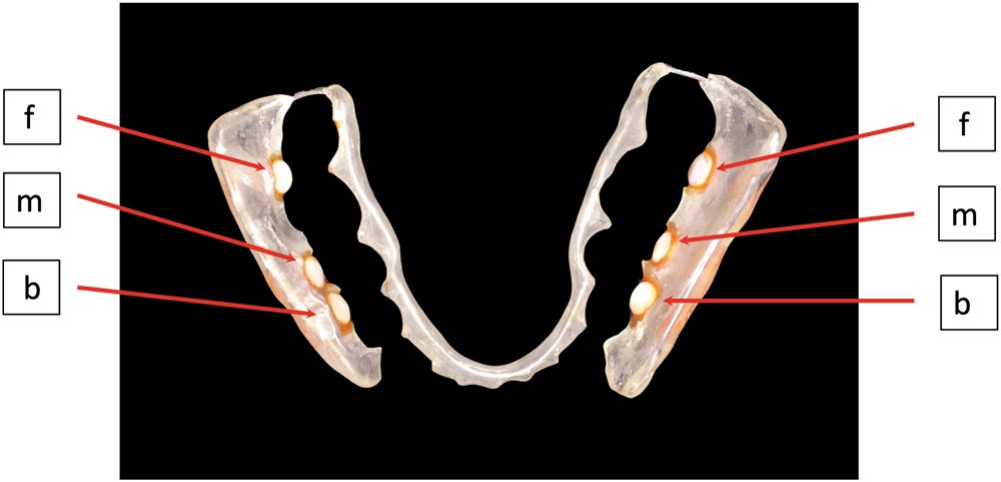
Splint system with bovine enamel slabs used to collect oral biofilm samples (f = frontal; m = mesial; b = buccal).

**Figure 2:**
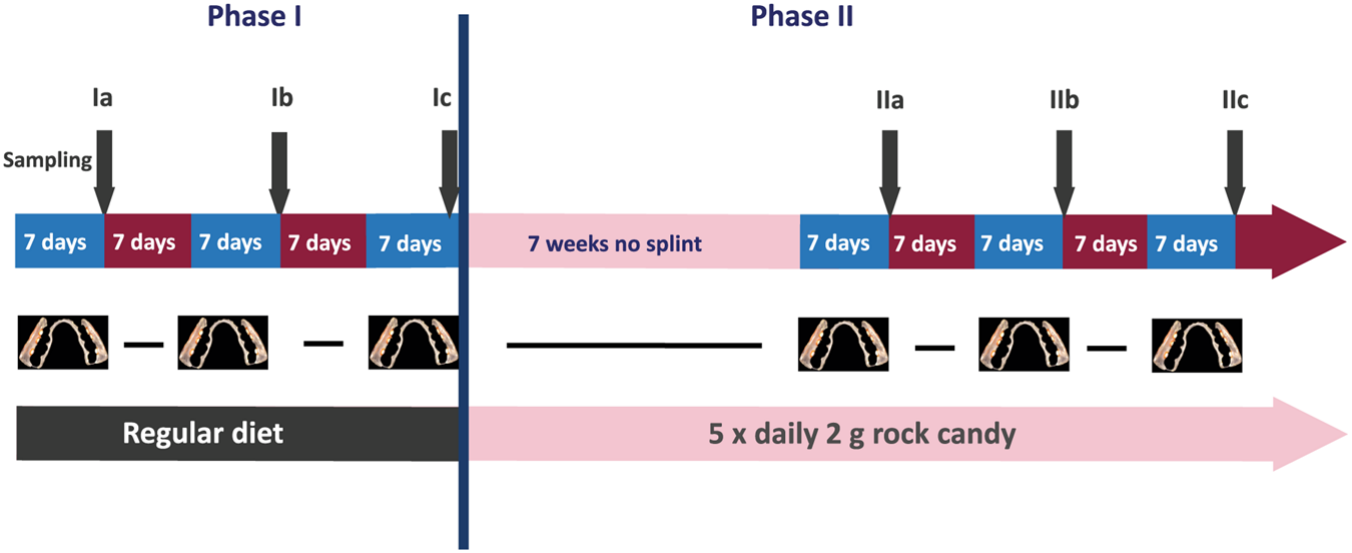
Study design. Samples of dental biofilm grown on enamel slabs embedded in splint systems were collected 3 times (**a**–**c**) in two phases (I, II). During phase I (grey), the 11 study participants kept their regular diet while in phase II (pink), frequent sucrose consumption was introduced for 3 months.

In this study we sampled dental plaque from splint systems (Fig. 1) worn by 11 study participants while keeping their regular diet (PI). Subsequently the participants changed to a three-month long diet with an additional daily consumption of 10 g sucrose in the form of small pieces of rock candy (2 g) 5 times between meals (PII). At the end of this phase the splint systems were worn again three times for seven days to obtain dental plaque samples (Fig. 2). The average age of the study participants was 32 years and their overall mean DMFT value was 8.1, none of them having open carious lesions. The demographic and clinical parameters of the study participants are shown in Table 1.

**Table 1:** Demographic and clinical data of study participants.

study participant	age	sex	DMFT-Index		PSI		Saliva flow rate [ml/min]		Buffering capacity	
			Phase I	Phase II	Phase I	Phase II	Phase I	Phase II	Phase I	Phase II
1	22	female	2	2	1-0-1-0-1-0	0-0-0-0-1-1	1.1	1.1	high	high
2	29	female	14	14	1-1-1-1-1-1	0-0-0-1-0-0	2.4	2.6	high	high
3	29	male	10	12	3-1-0-1-0-3	0-0-0-1-1-0	1.7	2.1	high	high
4	25	female	1	1	1-0-1-0-1-0	1-0-1-0-1-0	2.6	2.7	medium	medium
5	55	female	11	11	1-0-1-0-0-0	1-1-1-1-0-1	3.2	3.2	medium	high
6	21	female	8	8	1-2-2-2-2-2	1-2-2-2-2-2	1.6	1.7	high	high
7	28	male	14	14	2–2–2–2–2–2	—	1.4	1.7	medium	high
8	56	male	19	19	1-3-3-1-2-3	1-1-3-1-1-1	2.1	2.6	high	high
9	23	male	0	0	2-2-2-2-2-2	—	1.3	0.9	medium	medium
10	23	male	5	5	2-1-2-2-1-2	0-0-1-1-1-1	2.1	1.5	high	high
11	39	male	2	2	0-0-0-0-0-0	0-0-1-0-0-1	2.1	2.1	high	high

All study participants consumed a high carbohydrate western diet with over 45% carbohydrates as their regular diet^35^. The mean food frequency and amount of the main nutrients that the study participants consumed during phase I and phase II are listed in Table 2. The statistical analysis of the nutrition parameters did not reveal any significant differences regarding simple carbohydrate consumption in the regular diet of the participants during phases I and II ensuring that changes in the microbiota in PII can be attributed exclusively to the additional consumption of sucrose.

**Table 2:** Percentage of mean amount of main nutrients consumed by the 11 study participants in phase I and phase II. (The ratio of the additional 10 g sucrose in phase II referring to the percentage of simple CHO is included

Participant	Phase	Simple CHO	Ratio 10 g sucrose	Complex CHO	Total CHO	Protein	Fat
1	1	44.59%	—	24.98%	69.57%	14.91%	15.53%
	2	34.28%	4.63%	39.60%	65.72%	16.62%	17.66%
2	1	24.68%	—	45.35%	70.03%	17.15%	12.82%
	2	32.35%	5.17%	42.47%	65.90%	18.48%	15.62%
3	1	23.49%	—	33.98%	57.47%	21.01%	21.51%
	2	34.13%	2.23%	30.37%	60.31%	20.41%	19.29%
4	1	28.69%	—	28.94%	57.63%	20.65%	21.72%
	2	44.91%	3.56%	22.38%	60.69%	18.38%	20.92%
5	1	28.65%	—	36.28%	64.94%	17.35%	17.71%
	2	28.14%	6.87%	47.04%	64.14%	19.05%	16.81%
6	1	16.56%	—	35.97%	52.53%	24.84%	22.63%
	2	30.14%	2.05%	28.68%	54.99%	22.17%	22.85%
7	1	19.60%	—	31.15%	50.75%	24.79%	24.56%
	2	30.58%	6.65%	30.09%	49.74%	22.49%	27.77%
8	1	35.37%	—	27.67%	63.04%	18.50%	18.46%
	2	40.97%	2.40%	28.04%	64.48%	17.52%	18.01%
9	1	49.35%	—	22.01%	71.36%	13.17%	15.48%
	2	39.55%	3.57%	36.30%	69.30%	14.40%	16.30%
10	1	22.92%	—	38.70%	61.62%	19.10%	19.28%
	2	29.87%	3.80%	42.05%	65.18%	18.06%	16.76%
11	1	36.32%	—	28.32%	64.64%	20.49%	14.87%
	2	29.41%	5.73%	42.32%	61.86%	21.28%	16.87%
Total	1	30.02%	—	32.12%	62.14%	19.27%	18.58%
	2	34.03%	3.54%	35.39%	62.02%	18.99%	18.99%

.)

### Composition of the supragingival microbiota

**Figure 3:**
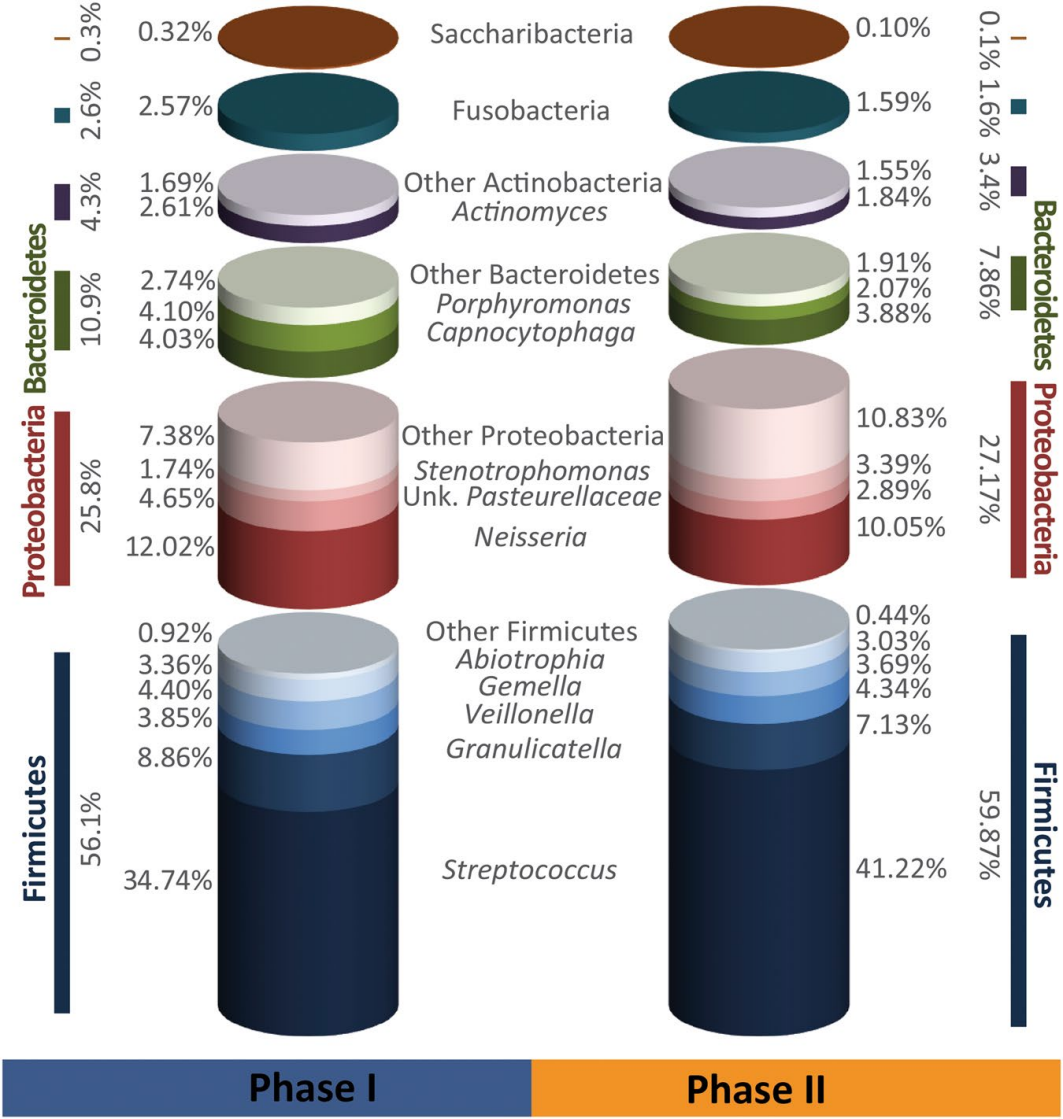
Relative average abundances (%) of the different phyla and the most abundant genera (>2% abundance among all samples) of the 11 study participants in phases I and II.

**Figure 4:**
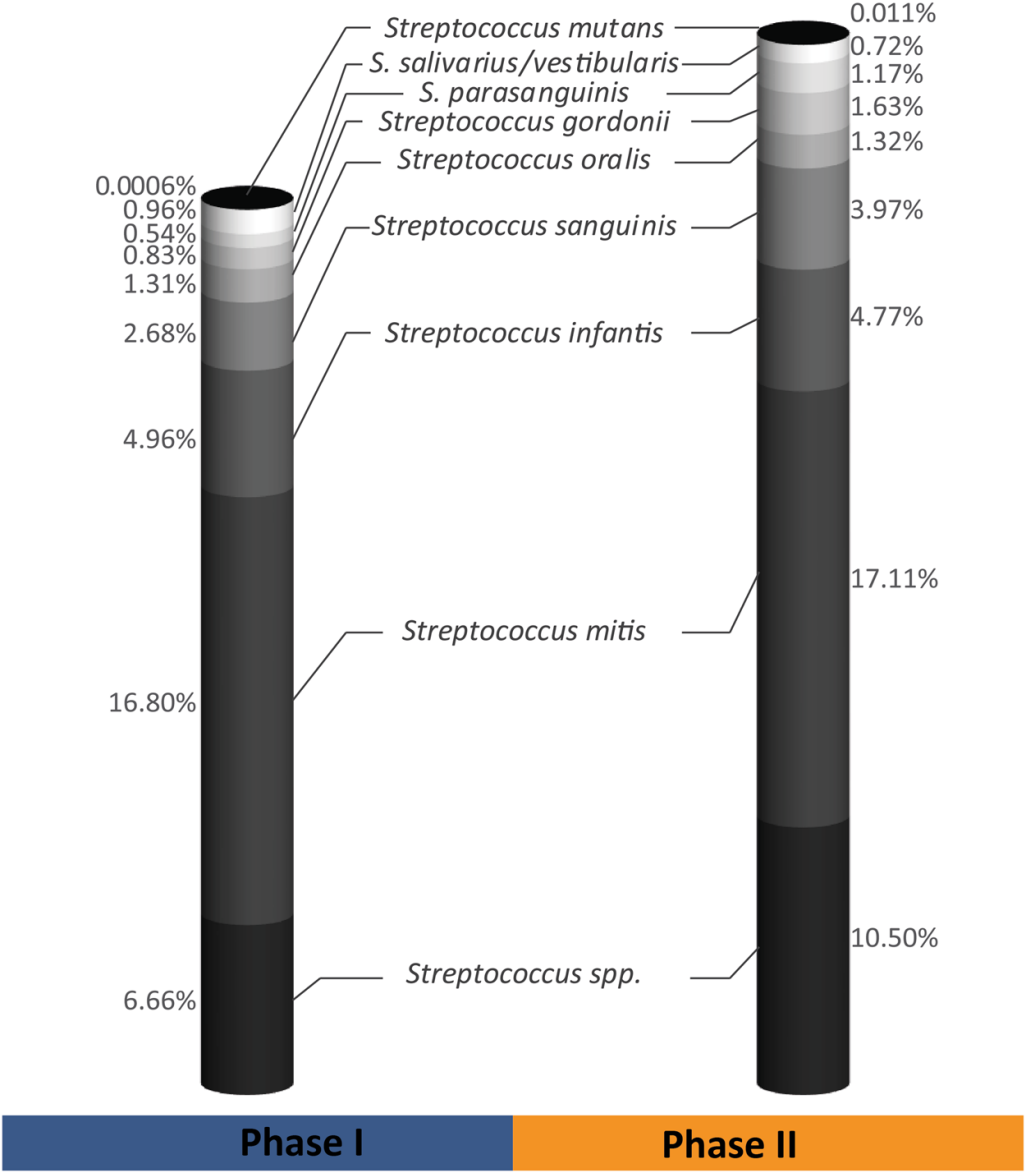
Relative average abundance (%) of the different *Streptococcus* species of the 11 study participants in phases I and II. *Streptococcus* spp. refers to those OTUs which could not be unambiguously assigned to one of the oral *Streptococcus* species.

Of the total number of 3.33 million high quality reads for the 66 samples of both dietary phases, 2.94 million sequences could be assigned to 225 species-level Operational Taxonomic Units (OTUs; 97% similarity). Abundances of the three sampling times in one phase were averaged to compare the two dietary phases. These OTUs belonged to six phyla with Firmicutes being the most abundant one, averaged 56.1% for PI and 59.87% for P II, followed by Proteobacteria, Bacteroidetes, Actinobacteria, Fusobacteria and Candidatus Saccharibacteria (former TM7) with an averaged abundance for PI/PII of 25.8/27.17%, 10.9/7.86%, 4.3/3.4%, 2.6/1.59% and 0.32/0.1% respectively as shown in Fig. 3. The species-level OTUs represented 59 taxa on the genus level. *Streptococcus* was the most abundant genus (34.74/41.22%) followed by *Neisseria* (12.02/10.05%), *Granulicatella* (8.86/7.13%), *Veillonella* (3.85/4.34%), *Gemella* (4.4/3.69%) and *Cytophaga* (4.03/3.88%) (Fig. 3; for details see Supplementary Figs S1 and S2). All of these taxa were present in both phases and showed 100% prevalence in the study participants. The full list of OTUs with their relative abundances is given in Table S3. For the genus *Streptococcus* a further allocation of the respective OTUs to individual species of oral streptococci was performed (Fig. 4). The highest average percentage was found for *S. mitis* (16.8/17.11%), followed by a group of OTUs which could not be further assigned to any *Streptococcus* species unambiguously (6.66/10.5%). *S. infantis* (4.96/4.77%), *S. sanguinis* (2.68/3.97%), *S. oralis* (1.31/1.32%), *S. gordonii* (0.83/1.63%) and *S. parasanguinis* (0.54/1.17%) were found in decreasing average percentages. The two species *S. salivarius* and *S. vestibularis* (0.96/0.72%) are presented in one group because they could not be further discriminated based on the sequenced fragment due to their high 16S rDNA similarity. Finally, *S. mutans* was the lowest abundant species of oral streptococci (0.0006/0.011%) and only detectable in a few study participants (phase I: 2/11 participants, phase II: 4/11 participants). The details of the species allocation and the abundances of the different *Streptococcus* species are shown in the distance matrix and the Supplementary Table S4 and Fig. S5.

### Species richness in supragingival plaque decreases and diversity changes during sucrose-rich diet

**Figure 5:**
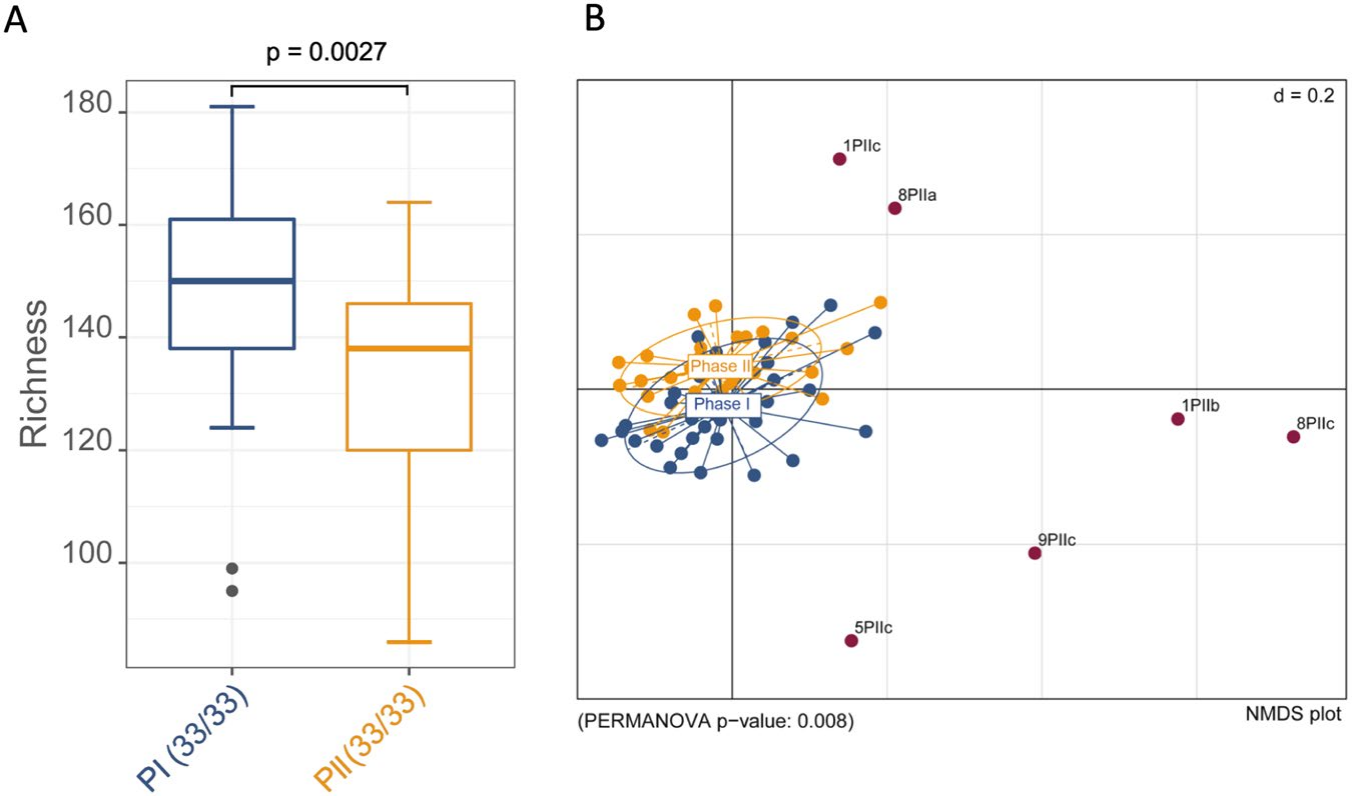
Species richness and beta-diversity in oral biofilm from 11 study participants in phases I and II. (**A**) Box plot depicting the decrease in species richness of all 33 samples in phase II. (**B**) NMDS plot depicting the beta-diversity in phases I and II. Samples from the second phase with radically altered microbial composition are displayed individually. Those outliers were mainly dominated by members of the phylum *Proteobacteria*.

A significant decrease of the species richness (p = 0.0027) from phase I to phase II (sucrose phase) was observed and is shown in Fig. 5A. The Shannon effective and Simpson effective as measures for the alpha-diversity show a decrease for 8/11 study participants which is significant for both indices (p = 0.020 and p = 0.033 respectively; Supplementary Table S6). The overall evenness decreased in phase II, but not significantly (Supplementary Table S6). At the same time, Permanova analysis revealed that the microbial communities in phase I and phase II were significantly different (p = 0.008); the beta-diversity analysis is shown in Fig. 5B.

### Shift of the microbiota during sucrose diet (phase II) – non-mutans streptococci increase significantly

**Figure 6:**
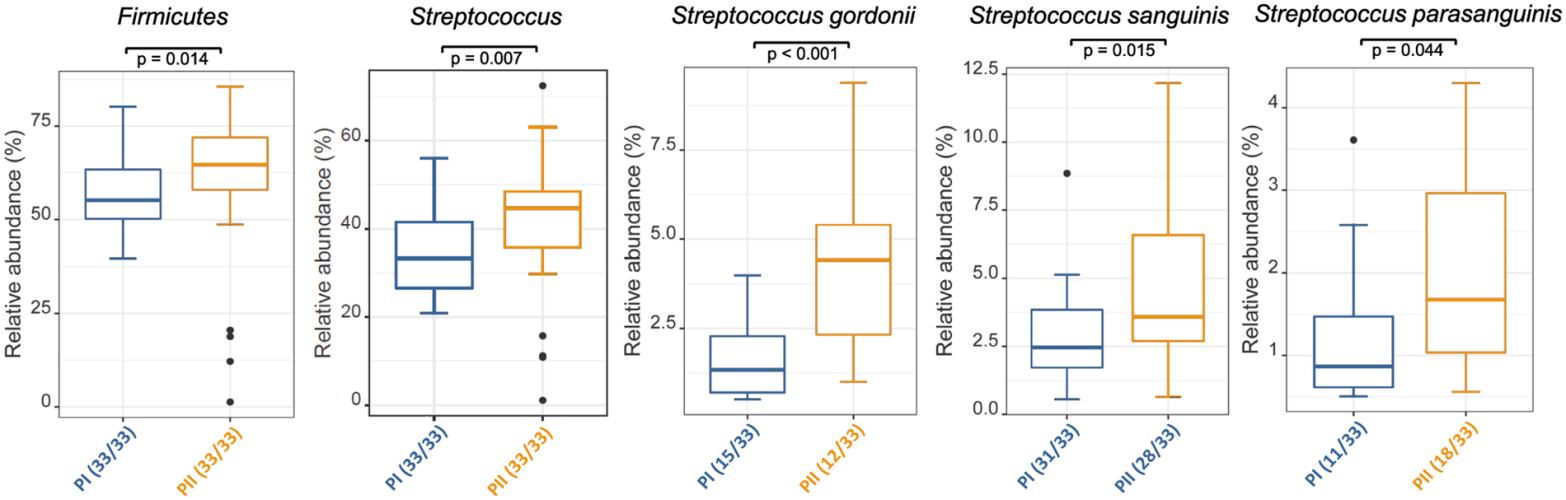
Box plots of the abundance of different taxa in oral biofilm that increase in phase II. The bottom and top of the box indicate the first and third quartiles, the line inside the box the median and the ends of the whiskers the 10th and 90th percentile values. Outliers are plotted as circles. Significant increases were detected in relative abundances of the phylum Firmicutes, the genus Streptococcus and the species S. gordonii, S. sanguinis and S. parasanguinis respectively.

In order to understand differences between the microbial communities in phases I and II serial group comparisons were performed in Rhea which revealed statistically significant differences for several taxa between phase I and II (shown in Fig. 6). We observed a significant increase of the phylum Firmicutes (p = 0.014) and the genus *Streptococcus* (p = 0.007) for all study participants. For the majority of the participants, several non-mutans streptococci increased significantly in phase II, i.e. *S. gordonii* (p = 0.001), *S. sanguinis* (p = 0.015) and *S. parasanguinis* (p = 0.044). Even though *S. mutans* was only found in two of the eleven participants and in very low abundances, it showed a substantial increase in phase II in these participants (phase I: 0.0006%; phase II: 0.011%).

**Figure 7:**
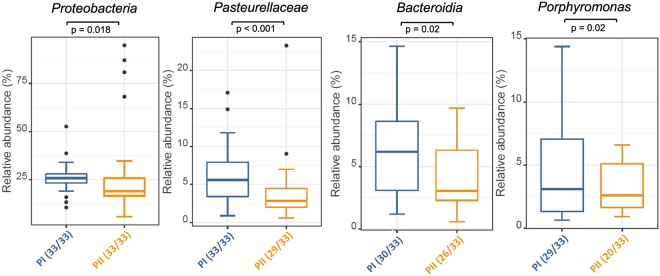
Box plots of the abundance of different taxa in oral biofilm that decrease in phase II. The bottom and top of the box indicate the first and third quartiles, the line inside the box the median and the ends of the whiskers the 10th and 90th percentile values. Outliers are plotted as circles. Significant decreases were found in relative abundances of the phylum Proteobacteria, the family Pasteurellaceae, the class Bacteroidia, and the genus Porphyromonas.

Several other taxa showed a significant decrease in abundance in phase II in either all, or at least the majority of the study participants (Fig. 7). The phylum Proteobacteria decreased overall (p = 0.018), however this change was only significant if four samples (1PIIb, 5PIIc, 8PIIc, 9PIIc) showing extraordinarily high abundances of Proteobacteria were treated as outliers. If the outliers were included, the average abundance of Proteobacteria even increased slightly in phase II but the change was not significant (cf with average abundance including outliers of Proteobacteria in Fig. 3). The family *Pasteurellaceae* decreased significantly (p < 0.001) with the main representative genera being *Haemophilus* and *Aggregatibacter*. Likewise a significant decrease of the class *Bacteroidia*, which was represented in the microbiota of the participants mainly by the genera *Prevotella* and *Porphyromonas* was observed, as well as of the genus *Porphyromonas* itself (p = 0.02 and p = 0.02 respectively).

### Changes in surface area roughness in phase II

The mean enamel surface roughness (Ra) over the entire period of phase I was 0.058 µm (95% CI: 0.052 µm; 0.068 µm). No significant differences were observed between the different weeks of phase I (week a (0.062 µm; 95% CI: 0.043; 0.081), week b (0.058 µm; 95% CI: 0.043 µm; 0.069 µm) and week c (0.058 µm; 95% CI: 0.05 µm; 0.062 µm)). In phase II mean surface roughness increased to 0.60 µm (95% CI: 0.057 µm; 0.062 µm), not significantly different from the value measured in phase I.

**Figure 8:**
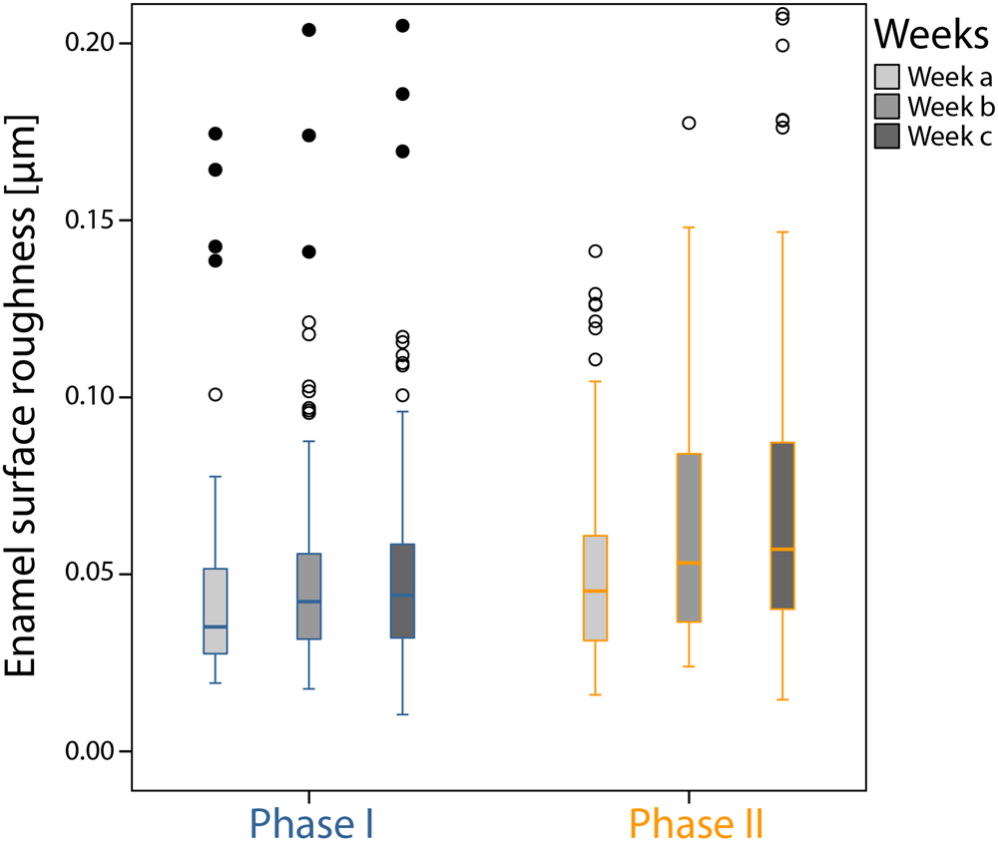
Arithmetic average of the roughness profile (Ra) [µm] of the enamel specimens for each week in phases I and II.

Regarding the individual weeks, the mean surface roughness increased in phase II (week a (0.050 µm; 95% CI: 0.047 µm; 0.052 µm), week b (0.059 µm; 95% CI: 0.056 µm; 0.063 µm) and week c (0.063 µm; 95% CI: 0.060 µm; 0.078 µm)), although not significantly. The changes are shown in Fig. 8.

## Discussion

Caries has been described as a primarily dietary-dependent disease that develops in the presence of oral microorganisms^36^. Apart from various factors, e.g. the texture of the consumed food or the contained nutrients, the amount and frequency of fermentable carbohydrates are believed to play the main role in caries development. Adler *et al*.^37^ interpreted their findings studying the changes in bacterial compositions of ancient dental calculus as reflecting two main dietary shifts in human history: the introduction of farming and the growing of cereals around 12.000 years ago, when the increased consumption of soft carbohydrates correlated with an increase in caries- and periodontitis-associated taxa. In the course of the Industrial Revolution (starting in the late 1700s) that boosted the availability of refined grain and sugar, caries-associated taxa again became more frequent in oral biofilm.

The aim of the present study was to show the influence of an individual temporary dietary shift towards frequent consumption of sucrose on the microbial community of the dental plaque of eleven volunteers wearing splint systems equipped with bovine enamel slabs. Additionally, we examined the effect of changes of the plaque microbiota on the enamel surface.

To the best of our knowledge this is the first study that uses the methodological benefits of high-throughput sequencing to analyse the change in plaque microbiota, grown *in situ*, brought about by an actual change in the amount and frequency of sucrose consumption of the study participants.

Rudney *et al*.^27^ tested the influence of sucrose pulses on plaque microcosms and found that five sucrose pulses daily clearly simulated caries-like pH drops in the microcosm biofilms and were able to infer a major role of non-mutans streptococci in lactate production. Based on these findings we can assume that in our situation, a five-time consumption of rock sugar was able to create similar pH drops which could drive the change of the abundance of non-mutans streptococci. Bradshaw and Lynch^2^ stated that especially frequent consumption of low molecular carbohydrates contributes to acid production. In the same way the present study has shown that the dietary change involving frequent sucrose consumption was the driving force towards a shift in the microbiota.

The most notable results that were observed consisted in the significant increase of the genus *Streptococcus* (p = 0.007) and several non-mutans *streptococci* in the majority of samples in phase II, (i.e. the sucrose-rich phase). In particular *S. gordonii*, *S. sanguinis* and *S. parasanguinis* increased significantly in the sucrose phase (p = 0.001, p = 0.015 and p = 0.044). The ecological plaque hypothesis postulates that changes in the environment lead to a shift in the microbiota where aciduric and acidogenic species proliferate and ultimately lead to the development of carious lesions^4^. The extension of this hypothesis takes into account the destabilization of the demineralization and remineralization balance towards a net mineral loss of the enamel caused by the involved acidogenic species^6^. When no mutans streptococci are present, the so called non-mutans streptococci can increase in the plaque microbiota and make it more acidogenic. Several studies deal with different acidogenic or ‘low pH’ non-mutans streptococci, e.g. *S. gordonii*, *S. oralis*, *S. mitis*, *S. anginosus*, *S. sanguinis* and characterize them as a heterogenous group that is able to adapt to acidic environment by changing physiological properties, and can increase their acidogenicity and acid tolerance^6^. Our findings confirm this hypothesis, as we find that several representatives of non-mutans streptococci were prevalent in all study individuals and their abundance was significantly elevated in the sucrose-rich phase.

The increase in abundance in the sucrose-rich phase was most significant for *S. gordonii*, an acidogenic species that is able to tolerate low pH^38,39^. De Soet *et al*.^40^ even reported strains of *S. gordonii* that were capable of producing acid at a similar rate as *S. mutans*, as long as the pH-value was kept between pH 5.0 and 5.5. In a study of the oral metagenome^41^, higher levels of *S. gordonii* were found in initial carious lesions than at advanced stages of caries. Gross *et al*.^12^ observed that although *S. gordonii* was present in individuals with carious lesions, it was detected more frequently when caries did not progress. Since we did not investigate the microbiota in already established carious lesions, and *S. gordonii* is known to degrade arginine to ammonia and thus elevate the pH again^42^, this species could possibly be more relevant to the shift of the microbiota at the beginning of caries onset than at later stages when other acidogenic species take over.

Next to *S. gordonii* we observed a significant increase in the abundance of *S. parasanguinis* in phase II. Different studies have associated this species with caries. Becker *et al*.^15^ found *S. parasanguinis* increased in progressed carious lesions in patients with early childhood caries (ECC). Gross *et al*.^12^ similarly reported a significant association of *S. parasanguinis* in their 16S rDNA cloning study in children with ECC, and Aas *et al*.^7^ detected *S. parasanguinis* frequently in carious primary and secondary teeth, especially when *S. mutans* was not present. Yet, in the same way as *S. gordonii*, this species is able to hydrolyze arginine to ammonia causing a pH rise. Hence it might also be associated more with the beginning stages of carious lesions.

The abundance of *S. sanguinis* also increased significantly in phase II. In their study of the acid tolerance and acidogenicity of different non-mutans streptococci, Takahashi and Yamada^43^ found that *S. sanguinis* was capable of increasing its acid production as well as its acid tolerance when exposed to sublethal preacidification at pH 5.5. *S. sanguinis* even continued to produce acid from glucose at a low pH of 4.0. In our study the frequent availability of sucrose increased the abundance of *S. sanguinis*. In a comprehensive profiling using a 16S rDNA cloning technique Peterson *et al*.^44^ reported a higher abundance of *S. sanguinis* in samples from caries-active individuals. Metagenomic studies likewise reported high percentages of this species in enamel and dentine caries^41,45^. However, other reports investigating later stages of caries have linked the detection of *S. sanguinis* to healthy sites rather than to carious lesions^15,46^. These conflicting findings might be explained by strain-specific differences that have been observed regarding acidogenicity and acid tolerance.

*S. mutans* was only found in two study participants and in very low abundances. In the sucrose phase its abundance in these two participants increased substantially and it was detected in samples of two more participants, yet altogether with abundances mostly below 0.01%. Some authors have found that amplifying the V1-V2 region of the 16S rDNA resulted in poorer detection of *S. mutans* compared to other primers^47,48^, while still achieving a better detection of the whole genus *Streptococcus*^49^. This is a possible reason for the low detection, yet even with culture technique, this species has not been detected frequently in our study participants (data not shown). However, low concentrations of *S. mutans* concur with other studies of the healthy supragingival plaque that were performed using primer systems for different regions^44,46,50,51^.

Nevertheless, in the individuals which harboured *S. mutans*, this species - even at low abundance levels - could interact with other streptococci and create synergies that could advance the disturbance of the homeostatic balance towards a more acidogenic microbial flora.

The genus *Actinomyces* has also been associated with the caries process and has been reported to be heterogeneous in regard to their acidogenicity. While less acidogenic representatives of this genus are present in higher abundances in the oral biofilm in health, the more acidogenic and aciduric strains increase in caries^52^. In this study, *Actinomyces* spp. were detected in both phases, yet at a lower abundance than in a comparable study^50^. Methodological reasons might be responsible for this result, i.e. the different experimental setup or the chosen DNA extraction method that did not include a mechanical lysis step. In addition the primer selection highly influences the high-throughput sequencing methods and it has been shown that the v1-v2 region, while suitable for the detection of *Streptococcus* species seems less suited to detect *Actinomyces* species^47^.

As far as *Lactobacilli* and *Bifidobacteria* are concerned, species of these genera are usually found in association with advanced caries and also more frequently associated with children than with adults^7,12,53^. As we investigated the supragingival microbiota of adults before the onset of caries we did not expect to find large numbers of these taxa. Low numbers of these taxa might have escaped detection due to methodological biases as mentioned before.

Several taxa were observed to decrease in the sucrose-rich phase. The genus *Porphyromonas* decreased significantly which is in agreement with studies that associated this species with a healthy, caries-free situation^7,44,50^. A recent study of the salivary microbiome found a strong association of the genus *Porphyromonas* with caries-free individuals in saliva samples^54^. Depending on the species, *Porphyomonas*, e.g. *P. gingivalis*, cannot tolerate acid, and is therefore likely to decrease in an environment where acidogenic and acid-tolerant species increase^42^.

The main representatives of the family *Pasturellaceae* (phylum *Proteobacteria*) which decreased significantly were the genera *Haemophilus* and *Aggregatibacter*. Different studies have found *Haemophilus* and the phylum *Proteobacteria* in general correlated with caries-free teeth, or a decrease of them during caries onset, respectively^12,44,46^. A potential caries-protective function of these and other taxa as well as their usefulness as indicators of caries-free situations deserve further research.

The overall composition of the supragingival microbiota of the study participants showed intra-individual differences, yet was clearly dominated by the phylum *Firmicutes*, in particular streptococci, followed by *Proteobacteria*, *Bacteroidetes*, *Actinobacteria*, *Fusobacteria* and *Candidatus Saccharibacteria* (formerly *TM7*). These findings are consistent with other studies investigating the supragingival plaque in healthy individuals^3,16,44,55^. In contrast to Eriksson *et al*.^50^ who analysed plaque microbiota of Swedish adolescents who - similar to Germans - are well educated as to caries prevention and have access to high quality dental care, we found a higher percentage of *Proteobacteria* than *Bacteroidetes*. The percentages of the other phyla differed as well. Apart from the fact that these authors did not use a splint system, this can be attributed to the fact that with high-throughput sequencing studies, the comparability of abundances of taxa between studies is restricted by many parameters introducing variability. Not only DNA-extraction methods, which were similar to our study, but also the primers that target different regions in the 16S rDNA gene as well as sequence analysis pipelines introduce considerable bias in high-throughput sequencing analyses, an obvious drawback of this approach when comparing different studies^47^. For our study, the data processing was performed using the IMNGS platform^56^ which applies the UPARSE analysis pipeline. UPARSE has been shown to provide high quality OTU classification when compared to other pipelines, while at the same time avoiding spurious OTUs^57^.

In phase II a significant decrease in richness as well as in alpha-diversity measured by the Shannon effective and the Simpson effective index value was observed. For the oral microbiota different findings have been reported concerning alpha-diversity in health and disease, e.g. Griffen *et al*.^18^ found a higher Shannon diversity in periodontitis than in healthy subgingival plaque. On the other hand, supragingival plaque samples showed a significant decrease in richness and diversity in carious compared to healthy teeth^8,44,58^. Gross *et al*.^12^ showed that the Shannon index values were lower the more caries progressed from intact enamel to cavitated lesions. Ribeiro *et al*.^59^ also found a lower Shannon diversity, although not significantly, in active white spot lesions compared to sound enamel, and observed an association of high consumption of fermentable carbohydrates with a reduction in diversity. Our observations in accordance with the aforementioned results support the extended ecological plaque hypothesis. The decrease in diversity can be attributed to the increase in acid produced by acidogenic non-mutans streptococci increasing in abundance. In the course of this process, species that do not tolerate the decline of the pH-value can be repressed and eventually eliminated, which might further advance the establishment of cariogenic plaque. Correspondingly, the analysis of the beta-diversity revealed that the microbial communities in the sucrose-rich phase were significantly different from phase I. Moreover, the number of outliers, i.e. samples which show a bacterial community composition distinctly different from all other samples in this study, strongly increased in phase II (Fig. 5B). This might point towards a general destabilization of the established plaque microbiome in these study participants following elevated sucrose consumption, which could provide not only chances for cariogenic, but also for other atypical microorganisms to proliferate in this environment.

The change in microbiota also had an effect on the enamel surface as was shown by the laserscanning microscope measurement. The surface roughness increased progressively towards the end of the sucrose-rich phase, even though this was not statistically significant. Nevertheless this observation is of importance as we had started out with healthy, caries-free study participants with healthy enamel. Duggal *et al*.^60^ analysed the demineralization of enamel slabs depending on the frequency of consumed sugar drinks. They showed that demineralization of enamel resulting in white spot lesions was observed even with a low frequency of sugar drinks (3 times daily) without fluoridated toothpaste, whereas with fluoridated toothpaste an effect was only visible when sugar drinks were consumed 7 or 10 times daily. A similar observation was made by Vale *et al*., studying the demineralization of enamel slabs rinsed with sucrose solution^61^. Using non-fluoridated toothpaste measurable demineralization occurred after 7 days. Our study participants were instructed to use fluoridated toothpaste as this is the common standard of dental hygiene in Germany. While brushing their teeth, the splint systems were taken out and the enamel slabs were not cleaned at any time. Still, it is possible that residual fluoride was still present in the oral cavity when the splint systems were put back in, since it has been shown that fluoride levels stay elevated for hours after toothbrushing^62^. Therefore a protective effect might have been possible, which is a weakness of the study. Nevertheless, a beginning impairment of the enamel surface was detected, and a study investigating an even longer-term dietary change might show the development of initial carious lesions.

One of the strengths of our study was an experimental design which took advantage of the actual complexity of the oral biofilm microbiota to investigate the direct influence of sucrose on its composition over a long period of time *in situ*. In contrast to other recent studies that used artificial plaque biofilm models, e.g. microcosms, in the present study we were able to investigate the actual diverse and complex plaque microbiota of the study participants^27,63,64^. The used splint system has proven to be suitable to cultivate the oral biofilm *in situ* and to investigate influences on the oral microbiota in previous studies^65–67^. The environmental parameters such as the availability of oxygen, the pH-value and the availability of nutrients correspond to the actual conditions of the oral cavity, even though the splint-system remains a clinical model system. However, the splint system in this study setup was indispensable since otherwise the required dietary change and simultaneous omission of oral hygiene would have been unethical considering the participants’ dental health. To ensure that the increased sucrose consumption did not have any adverse effects on the study participants dentition, only study participants with a sufficient salivary flow rate and buffer capacity, adequate oral hygiene (flossing) and a restored dentition were included in the study, criteria that are adequate for the protection of their oral health. The participants visited the clinical study center regularly to have their oral health monitored and clinical consequences, i.e. white spots, could not be detected after the end of phase II.

Combining this splint system approach with high-throughput sequencing, which is capable of detecting not yet cultured species, we were able to directly monitor the influence of frequent sucrose consumption on the microbial community.

In conclusion, in monitoring the changes of the microbial community in dental plaque provoked by frequent consumption of sucrose following a dietary change, our study corresponds with the extended ecological plaque hypothesis and highlights the polymicrobial synergy of non-mutans streptococci in the early stages of caries onset. The observed changes in the microbiota were reflected in the increase of the enamel surface roughness. Our results attributed a major role to the increased abundance of non-mutans streptococci, and demonstrated that significant changes in the abundance and diversity of the microbiota came about after a relatively short-term dietary change. Even though enamel changes were not significant after such a short time, sucrose frequency restriction still appears to be the most important preventive measure against caries development. Future research should focus on metagenomic approaches to further reveal what takes place on a functional level within the microbial community of the oral biofilm.

## Material and Methods

### Study group

The study protocol was approved by the Ethics Committee of the University of Freiburg (Nr. 237/14). All experiments and data collections were performed in accordance with relevant guidelines and regulations. Eleven healthy volunteers, aged between 21 and 56 participated in the study after giving their written informed consent. Exclusion criteria for the study were: severe systemic disease, diseases of the salivary glands or the oral mucosa, diabetes, use of antibiotics or local antimicrobials or fluoridated mouth rinses within the last 30 days, current dental treatment, eating disorders, food intolerances or food allergies, pregnancy or lactation. Prior to the study phases, a clinical oral examination was performed and DMFT (decayed, missing, filled teeth) values were collected. Additionally, the salivary flow rates and its buffering capacity were measured (Table 1). Each study participant received a standardized tooth brush (Friscodent, Aldi Süd, Germany) and a customary toothpaste with a sodium fluoride content of 1.450ppm (Friscodent, Aldi Süd, Germany).

### Dietary change and diet assessment

The dietary phases and the periods when the splint systems were worn are shown in Fig. 2. After adjustment of the individual splint systems, each study participant was instructed to keep their regular diet and wear the splint system for 3 × 7 days with seven-day intermissions to obtain dental biofilm samples. The splint system was worn at all times except during meals and dental hygiene. When it was taken out, it was kept in 0.9% NaCl. After this first phase (PI), each study participant was instructed to keep his regular diet and additionally suck 10 g rock candy (Weisser Kandis, Südzucker AG, Mannheim, Germany) in small pieces of 2 g five times between meals. The dietary change with the sucrose consumption (PII) lasted for three months and at the end of this phase, the participants wore the splint systems again for 3 × 7 days with seven-day intermissions to obtain dental biofilm samples from the enamel slabs and equip the splint system with new enamel slabs. During these weeks, the splint system was also worn while the participants were sucking the rock candy, yet was taken out during regular meals.

The basic diet of the participants was monitored during both phases using a validated food frequency questionnaire which assessed the average dietary behavior for one month retrospectively^68^. The additional consumption of sucrose during phase II was monitored with a form that the participants had to complete to check the required daily consumption of the rock candy. Statistical analysis using paired t-test was performed to compare the nutrition parameters of the basic diet during the two phases.

### Bovine enamel slabs (BES) and splint systems

The bovine enamel specimens used for the collection of dental biofilm in the dental splint systems were prepared as described in earlier studies^67^. In brief, cylindrical enamel slabs (5 mm diameter, 1 mm height and 19.63 mm^2^ surface area) were punched out of the labial surfaces of bovine incisors after confirming the bovine spongiform encephalopathy (BSE)-free status of the cattle. The enamel surfaces were polished by wet grinding with abrasive paper (220 to 4000 grit, Knuth-Rotor-3, Streuers, Willich, Germany). Subsequently the specimens were disinfected using ultrasonication with 3% NaOCl for 3 min followed by air drying and an additional ultrasonication in 70% ethanol. After disinfection, the specimens were ultrasonicated twice in double-distilled water for 10 min and stored in aq. dest. for 24 h until use. Lower jaw acrylic appliances were prepared as splint systems for each study participant and loaded with six bovine enamel slabs (BES) in the interdental area between upper premolars and molars, so that the biofilm formation could take place undisturbed by movements of the tongue (Fig. 1). After each test phase, the BES and the adherent dental biofilm were removed and used for the high-throughput sequencing analysis. For the subsequent surface roughness analysis, the enamel surfaces were thoroughly cleansed using cotton pellets and saline solution.

### Sample collection and DNA extraction

The splint systems with adherent dental biofilm were rinsed with NaCl to remove cells that had not adhered to the BES. For the high-throughput sequencing method, dental biofilm formed on the six BES was removed with a sterile soft plastic spatula and put in a vial containing 950 µl RTF medium^69^. The vial was vortexed and ultrasonicated for 1 min at 70% power (Bandelin, Berlin, Germany) and kept at −80 °C until use. From this suspension, 300 µl were taken for DNA extraction. For the surface roughness measurement, two BES were air dried and stored in an Eppendorf vial.

The DNA was extracted using the DNeasy blood & tissue Kit (Qiagen, Hilden, Germany) according to the manufacturer’s protocol for bacteria including the pretreatment steps for Gram-positive bacteria. Enzymatic lysis was performed using 150 µl enzymatic lysis buffer containing 20 µl lysozyme (20 mg/ml) and 30 µl mutanolysin (1.500 U/ml; Sigma Aldrich, Taufkirchen, Germany) for 1.5 h at 37 °C. The Proteinase K incubation was extended to 1.5 h at 56 °C. Microbial DNA was eluted twice in 100 µl AE buffer and then stored at −20 °C.

### Illumina MiSeq high-throughput-sequencing

The microbial community in the dental biofilm was analysed using Illumina MiSeq paired-end-sequencing with two times 300 bp read length. For the amplicon library the primer pair S-D-Bact-0008-a-S-16 (5′-AGA GTT TGA TCM TGG C-3′) and S-D-Bact 0343-a-A-15 (5′-CTG CTG CCT YCC GTA-3′) amplifying a 335 bp fragment of variable regions v1-v2 and including the recommended adaptors for Illumina sequencing was used. PCR was optimized resulting in 23 cycles and 1 ng of template DNA per 25 µl reaction. The NEBNext^®^ High-Fidelity 2X PCR Master Mix (New England Biolabs, Frankfurt, Germany) was applied for amplification and PCR was set up according to the manufacturer’s recommendation. PCR cleanup and removal of small fragments was done with the NucleoSpin Gel and PCR Clean-up Kit (Macherey-Nagel, Düren, Germany) using a 1:4 diluted binding buffer NTI. With a 2100 Bioanalyzer and a DNA 7500 chip (Agilent, Waldbronn, Germany) the size of the target fragment and the absence of small fragments were verified. Exact quantification was performed with a PicoGreen assay (QuantIT, Thermo Fisher Scientific, Schwerte, Germany). Indexing, pooling and sequencing were performed following the Illumina MiSeq protocol for amplicon sequencing. For 66 samples this resulted in a total of 3.33 million reads after joining forward and reverse reads.

Data processing was performed using the IMNGS platform^56^ applying the UPARSE amplicon analysis pipeline^57^. Statistical evaluation was done with the Rhea pipeline for R^70^. The species level analysis for the OTUs allocated to the genus *Streptococcus* was done by manually extracting all sequences of OTUs assigned to streptococci and performing a phylogenetic analysis with the ARB software package^71^. Via a distance matrix all OTUs were identified which showed a clear association with only one of the oral *Streptococcus* species, i.e. a 16S rDNA sequence similarity of 97% or higher with the species *S. infantis*, *S. mitis*, *S. oralis*, *S. gordonii*, *S. sanguinis*, *S. parasanguinis*, *S. salivarius/vestibularis* and *S. mutans*. If there were ambiguous affiliations (higher than 97% similarity to more than one species, or to none) or affiliations with other species the OTU was not further allocated and grouped as *Streptococcus* spp.

### Analysis of the surface roughness of the enamel

After thoroughly cleaning the surface of the slabs, the enamel surfaces were visually checked for any remnants using the camera unit of the Keyence 3D Laserscanning Microscope VK-X210 (Keyence Deutschland GmbH, Neu-Isenburg, Germany). To measure the surface roughness, a polygonal measuring field was selected. Ideally, the complete surface was measured with a resolution of 1000/mm, λS = 2.5 and λc = 0.25. In cases of mechanical damage to the specimen (e.g. break-offs at the specimen’s edges) those areas were excluded from the measurement.

### Statistical analysis

Statistical analysis was done with the Rhea package for R (s. above). Briefly, data were normalized and from this alpha and beta-diversity were calculated. For the latter generalized UniFrac was used. Visualization of the multidimensional distance matrix in a space of two dimensions is performed by Multi-Dimensional Scaling, or its more robust and unconstrained non-metric version (NMDS). This was followed by a taxonomic binning and a serial group comparison. For more details see the script available in the download package of Rhea. Additionally, linear mixed models with random intercepts were used to analyse differences in the parameters Shannon effective and Simpson effective in phases I and II. The nutrition parameters in the two different phases were compared with a paired t-test.

## Electronic supplementary material

Supplementary Figures

Dataset 1

## Data Availability

The datasets supporting the conclusion of this article are available through GenBank (file SUB3604614: accession numbers MG875094–MG875318) and can be found at: www.ncbi.nlm.nih.gov/popset/?term=1337416668.
