# Supplementary material for: In-vivo shift of the microbiota in oral biofilm in response to frequent sucrose consumption
Source: Sci Rep. 2018 Sep 21;8:14202. doi: 10.1038/s41598-018-32544-6 (PMC6155074; doi:10.1038/s41598-018-32544-6)

Supplementary Info File

In-vivo shift of the microbiota in oral biofilm in response to frequent sucrose consumption

Annette Carola Anderson[1*+], Michael Rothballer[2+], Markus Jörg Altenburger[1], Johan Peter Woelber[1], Lamprini Karygianni[3], Ilias Lagkouvardos[4], Elmar Hellwig[1], Ali Al-Ahmad[1]

[1]Department of Operative Dentistry and Periodontology, Faculty of Medicine, Albert-Ludwigs-University, Freiburg, Germany

[2]Institute of Network Biology, Helmholtz Zentrum München, German Research Center for Environmental Health, Neuherberg, Germany

[3]Clinic of Preventive Dentistry, Periodontology and Cariology, Center of Dental medicine, University of Zürich, Switzerland

[4]ZIEL - Institute for Food and Health, Core Facility Microbiome/NGS, Technical University of Munich , Freising , Germany

[+]ACA and MR contributed equally to this work

Address correspondence to:

Dr. Annette Anderson
Department of Operative Dentistry and Periodontology,
Hugstetter Straße 55
79106 Freiburg
Germany
annette.anderson@uniklinik-freiburg.de

Figure S1: **Relative abundances**

Relative abundances (%) of the different phyla found in all samples from the 11 study participants.

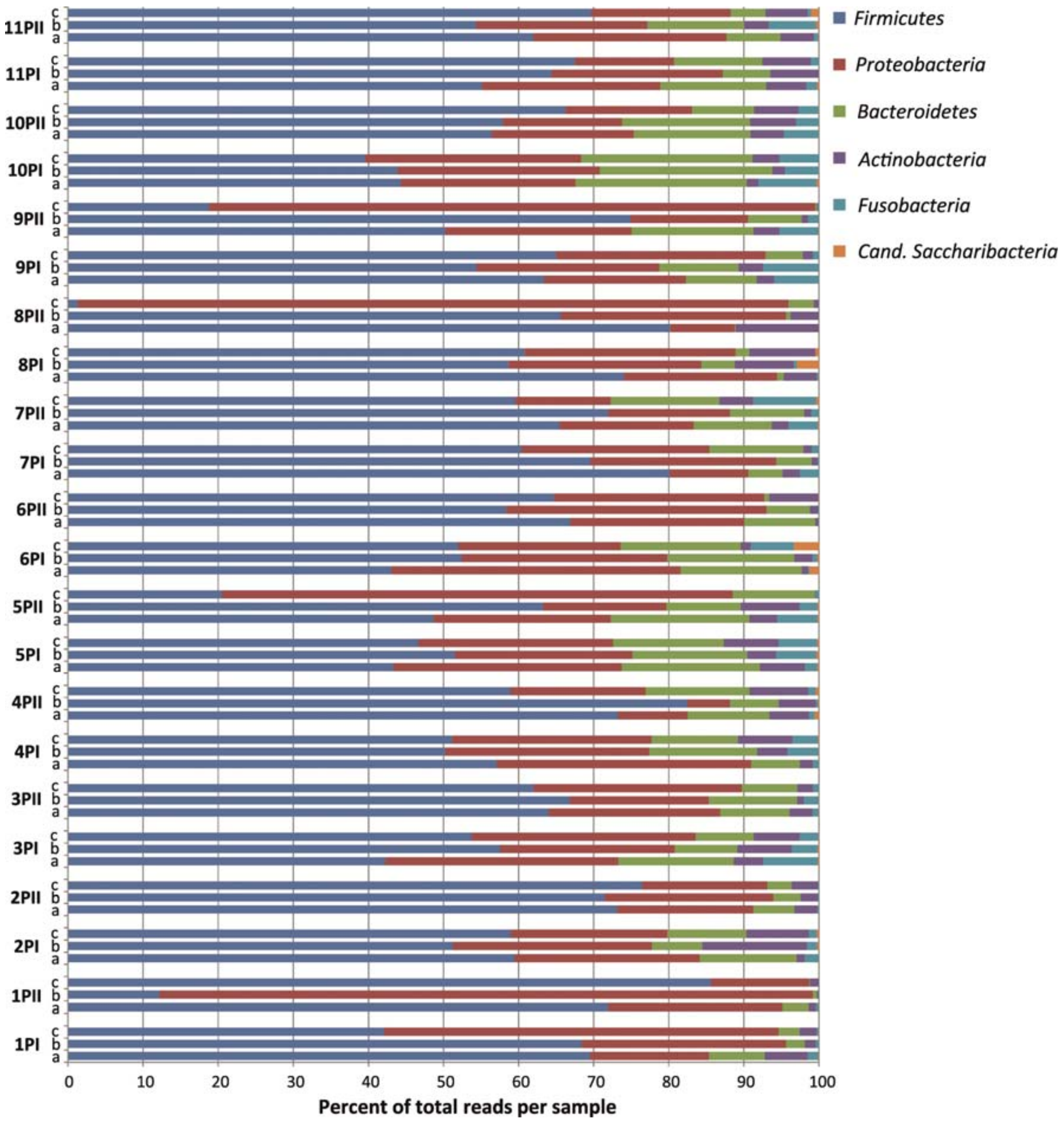

Figure S2: **Relative abundances**

Relative abundances (%) of the most abundant genera (> 2% abundance) found in all samples from the 11 study participants.

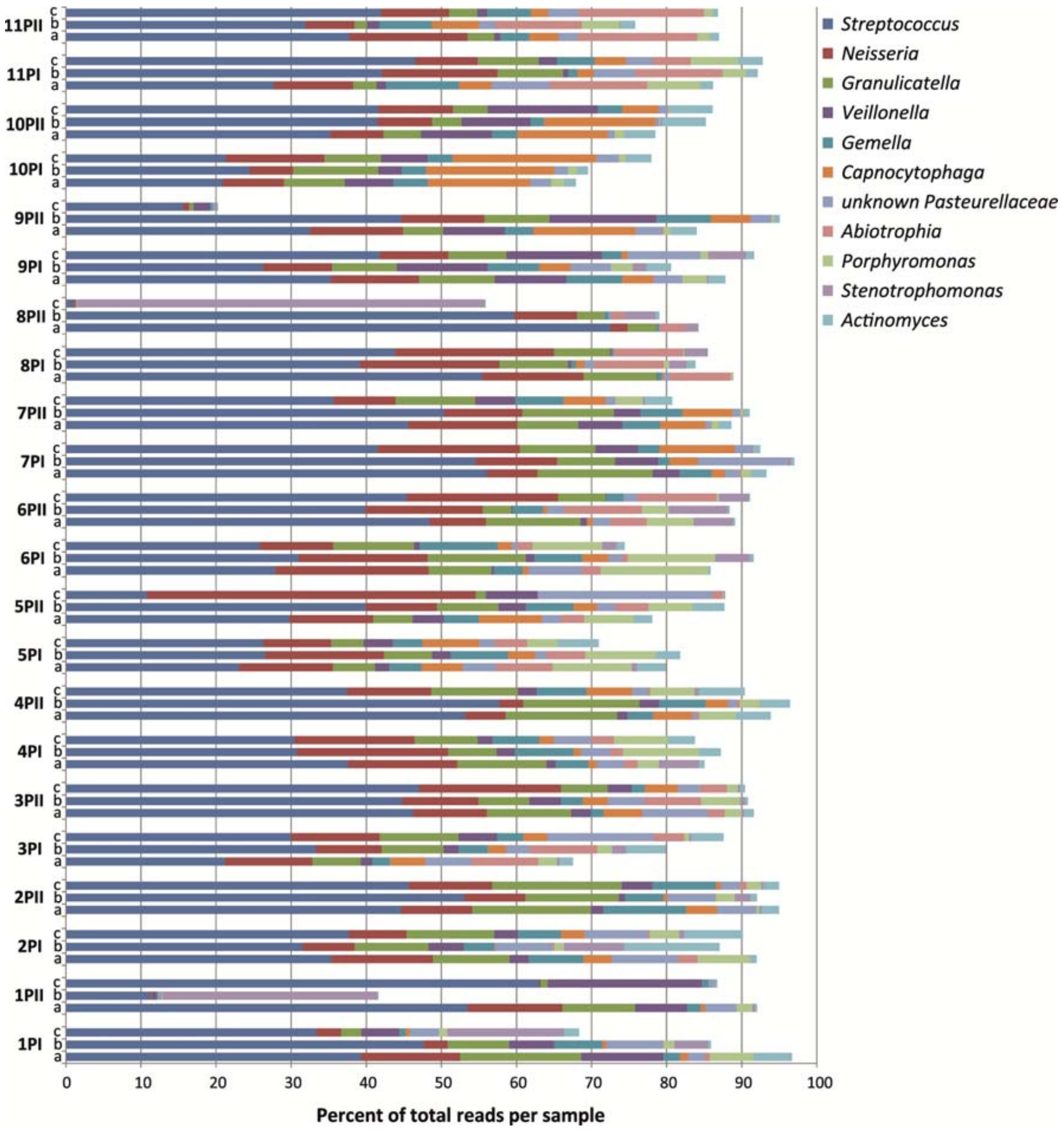

Figure S5: Abundances of *Streptococcus* spp.

Relative abundances of the different *Streptococcus* species of all samples from the 11 study participants. *Streptococcus* spp. refers to those OTUs which could not be unambiguously assigned to one of the oral *Streptococcus* species.

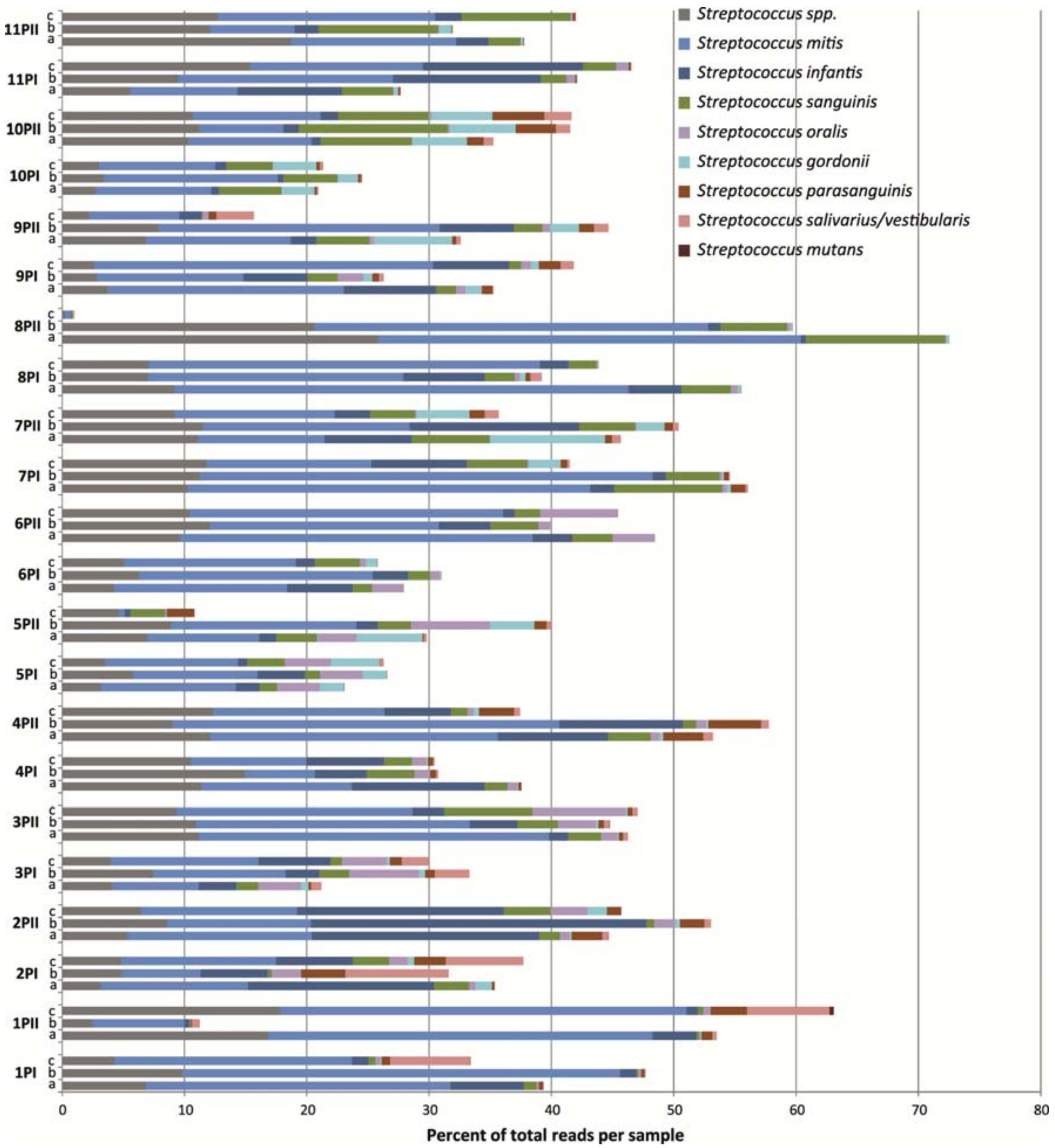

Supplement: Supplementary file 1 — Supplementary Figures [file 41598_2018_32544_MOESM1_ESM.pdf]
